# Supplementary material for: Plasmalogen loss caused by remodeling deficiency in mitochondria
Source: Life Sci Alliance. 2019 Aug 21;2(4):e201900348. doi: 10.26508/lsa.201900348 (PMC6707388; doi:10.26508/lsa.201900348)
Supplement: Supplementary file 1 [file LSA-2019-00348_Supplementary_Text_1.doc]

Appendix 1

Only plasmenylcholine but not plasmenylethanolamine is lost in the TAZ-KD mouse heart

One of the puzzling observations we have encountered in our previous work associated with the dramatic loss of plasmenylcholine in the TAZ-KD mouse heart was no significant change in the plasmenylethanolamine level (Kimura et al., 2018) (Fig. 2A). As plasmenylethanolamine is formed on the synthesis pathway leading to the production of plasmenylcholine (Fig. S2), no change in the plasmenylethanolamine level implied that there is no defect in the mechanism of plasmalogen synthesis unlike the cases of other diseases that are recognized with a plasmalogen loss like Zellweger syndrome (Heymans et al., 1983) and rhizomelic chondrodysplasia punctata (RCDP) (Dorninger et al., 2015). Both choline and ethanolamine plasmalogens are deficient in Zellweger syndrome due to the global defect in biogenesis of peroxisomes and in RCDP due to deficiency in one of the peroxisomal enzymes related to synthesis of plasmalogen precursors (Wanders and Brites, 2010).

In fact, quantitative Western blotting experiments revealed the absence of any losses of Pex19p and PMP70 in the TAZ-KD heart (Kimura et al., 2018): Pex19p is a protein that plays a critical role in the *de novo* formation of the peroxisomal membrane (Fujiki et al., 2014; Purdue and Lazarow, 2001; Smith and Aitchison, 2013), and PMP70 is a peroxisomal membrane protein that is often used as a marker to evaluate the amount of peroxisomes (Sugiura et al., 2017; Uyama et al., 2015). In addition, expression of fatty acyl-CoA reductase 1 (Far1), a rate determining enzyme of plasmalogen synthesis that produces fatty alcohol from fatty acid (Fig. S2), was dramatically upregulated by 8.3 (± 1.6)-fold to accelerate plasmalogen synthesis in response to the reduction in the plasmenylcholine level (Kimura et al., 2018) (Fig. 3B). The expression level of Far1 is known to respond to the plasmalogen level by a feedback mechanism (Honsho et al., 2010). Therefore, the observed lack of change in the steady state level of plasmenylethanolamine in the heart is likely to be merely a reflection of a balance between the accelerated plasmalogen synthesis caused by the Far1 upregulation, and a conceivably present decrease of plasmenylethanolamine by a mechanism analogous to that for the decrease of plasmenylcholine. Those ^31^P NMR and the Western blotting results together contributed to reaching the hypothesis presented in the introduction section, i.e., plasmalogen losses in other organs, tissues, and cells where plasmenylethanolamine is the dominant form of plasmalogen.
