# Supplementary material for: Plasmalogen loss caused by remodeling deficiency in mitochondria
Source: Life Sci Alliance. 2019 Aug 21;2(4):e201900348. doi: 10.26508/lsa.201900348 (PMC6707388; doi:10.26508/lsa.201900348)
Supplement: Supplementary file 2 [file LSA-2019-00348_Supplementary_Text_2.doc]

Appendix 2

Mechanism of the counterbalance of a plasmalogen loss by a gain of the counterpart diacyl glycerophospholipid

In Zellweger syndrome and RCDP there is a defect in synthesis of plasmalogen precursors, which in the normal case should take place in peroxisomes upstream of the following synthesis steps to evolve and complete in the ER (Fig. S2) (Wanders and Brites, 2010). Synthesis of plasmenylethanolamine and that of diacyl PE likely compete in the ER typically on the enzymatic ethanolaminephosphotransferase (EPT) activity for reaction with cytidine diphosphate (CDP)-ethanolamine (Fig. 1C). This competition will be by their respective precursor substrates 1-*O*-alkyl-2-acyl-glycerol (a precursor leading to plasmenylethanolamine via plasmanylethanolamine) and 1,2-diacyl-glycerol. Similarly, synthesis of plasmenylcholine and that of diacyl PC are considered to compete in the ER typically on the enzymatic cholinephosphotransferase (CPT) activity for reaction with CDP-choline by their respective precursor substrates 1-*O*-alk-1′-enyl-2-acyl-glycerol and 1,2-diacyl-glycerol (Fig. 1C). A counterbalancing increase of a diacyl glycerophospholipid observed in Zellweger syndrome and RCDP may be explained by those competitions on the EPT and CPT activities, compensating for a loss of the substrate for plasmalogen synthesis due to an upstream defect related to the integrity of peroxisomes (Dorninger et al., 2015).

In the case of the TAZ-KD mouse heart, observations of (i) the normal steady state level of plasmenylethanolamine which is a precursor in plasmenylcholine synthesis (Fig. 2A; Fig. S2; and Appendix 1) and (ii) acceleration of plasmalogen synthesis by upregulation of a rate determining enzyme Far1 (Kimura et al., 2018) (Fig. 3B; Fig. S2; and Appendix 1) in response to the reduced plasmenylcholine level, likely indicate the absence of a reduction in the substrate supply to the CPT activity. The counterbalancing gain of diacyl PC is explained by feedback regulation by the two products to this enzymatic activity, sensing a loss of one of the products (plasmenylcholine) and compensating for this loss by an increased concentration of the other product (diacyl PC) (Fig. 1C).

The counterbalance of a plasmenylethanolamine loss by a gain of diacyl PE observed in other TAZ-KD mouse organs and in the lymphoblast of BTHS patients, where ethanolamine is the dominant class of plasmalogen, may be similarly explained by feedback regulation by the two products to the EPT activity, i.e., plasmanylethanolamine and diacyl PE (Fig. 1C; and Fig. S2); a loss of plasmenylethanolamine causes feedback regulation on plasmanylethanolamine desaturase to possibly result in a loss of the substrate plasmanylethanolamine. The normal levels of peroxisomes as well as the rate-determining Far1 enzyme as evaluated in the case of BTHS lymphoblast (Fig. 3, A and B) suggest the normal rate of the substrate supply to the EPT activity, in favor of the feedback regulation as the mechanism of the counterbalance.
