# Supplementary material for: Plasmalogen loss caused by remodeling deficiency in mitochondria
Source: Life Sci Alliance. 2019 Aug 21;2(4):e201900348. doi: 10.26508/lsa.201900348 (PMC6707388; doi:10.26508/lsa.201900348)
Supplement: Supplementary file 3 [file LSA-2019-00348_TableS1.docx]

| **Table S1. Contents and Changes (mol %)^1^ of**  **the Brain Phospholipids of the WT versus TAZ-KD Mice** | | | | | |
| --- | --- | --- | --- | --- | --- |
| phospholipid | chemical shift^2^ (ppm) | content | | change in contribution to the total phospholipid | change in terms of the individual content |
|  |  | WT | TAZ-KD | TAZ-KD ‒ WT | [(TAZ-KD ‒ WT)/WT]×100 |
| diacyl PC (with plasmanylcholine)^3^ | ‒0.188 | 36.9 ± 0.5 | 37.0 ± 0.5 | +0.1 ± 0.7 | +0.2 ± 1.8 |
| plasmenylcholine | (‒0.177)^5^ | N.R.^6^ | N.R.^6^ | N.A.^8^ | N.A.^8^ |
| PI | 0.026 | 2.8 ± 0.3 | 3.1 ± 0.2 | +0.3 ± 0.3 | +10.3 ± 12.2 |
| PS | 0.119 | 11.5 ± 0.9 | 12.5 ± 0.2 | +0.9 ± 1.0 | +8.2 ± 8.4 |
| lyso PC | 0.191 | 0.3 ± 0.1 | 0.2 ± 0.0 | ‒0.1 ± 0.1 | ‒34.1 ± 22.8 |
| diacyl PE (with plasmanylethanolamine)^3^ | 0.295 | 16.0 ± 0.8 | 16.7 ± 0.7 | +0.7 ± 1.1 | +4.6 ± 6.9 |
| plasmenylethanolamine | 0.336 | 26.2 ± 0.9 | 23.5 ± 0.5 | ‒2.8 ± 1.1 | ‒10.5 ± 4.1 |
| SM | 0.371 | 3.7 ± 0.9 | 4.1 ± 0.4 | +0.4 ± 0.9 | +12.1 ± 25.6 |
| lyso PE | 0.656 | 0.1 ± 0.0 | 0.1 ± 0.0 | 0.0 ± 0.0 | ‒38.6 ± 26.9 |
| CL | 0.735 | 0.6 ± 0.4 | 0.7 ± 0.1 | +0.1 ± 0.4 | +18.3 ± 66.6 |
| PG | 0.831 | 0.2 ± 0.1 | 0.4 ± 0.0 | +0.2 ± 0.1 | +139.8 ± 60.7 |
| 1-MLCL | 0.985 | N.D.^7^ | 0.0 ± 0.0 | 0.0 ± 0.0 | N.A.^8^ |
| 2-MLCL | 1.115 | N.D.^7^ | 0.1 ± 0.0 | +0.1 ± 0.0 | N.A.^8^ |
| PA^4^ | 4.104 | 1.7 ± 0.1 | 1.6 ± 0.1 | ‒0.1 ± 0.2 | ‒4.6 ± 9.4 |
| ^1^The average and error, shown as the standard deviation, are obtained from three independent biological samples (N = 3) for each of the WT and TAZ-KD mice.  ^2^Values are from observation at 25°C in 10% (w/v) SDS micellar solution at pH=8.5 [50 mM EPPS, 50 μM BHT, 10% (v/v) D_2_O], in reference to the diacyl PE (with plasmanylethanolamine) peak set to 0.295 ppm as an internal standard (Kimura et al., 2018).  ^3^The signal of the plasmanyl glycerophospholipid as a minor component overlaps with the signal of the counterpart diacyl glycerophospholipid (Kimura et al., 2018).  ^4^Notable amounts of this lipid were detected in the brain by ^31^P NMR, and were measured at pH=8.5 shifting its resonance downfield to avoid overlap with MLCL signals (Fig. S4).  ^5^Value estimated based on the difference from that of diacyl PC (with plasmanylcholine) (Kimura et al., 2018). The absolute chemical shift values of the extracted phospholipids slightly depended on the organ and cell type, likely because of the presence of some degree of co-extracted materials that depend on the organ/cell type and may hence influence differentially the molecular environment of the phosphate of the same phospholipid class or subclass. The standard deviation of measured chemical shift values of diacyl PC (with plasmanylcholine) in the lipid extract from the brain tissue was 0.001 ppm.  ^6^Signal not resolved.  ^7^Not detected.  ^8^Not applicable.  **Reference**  Kimura, T., A.K. Kimura, M.D. Ren, B. Berno, Y. Xu, M. Schlame, and R.M. Epand. 2018. Substantial decrease in plasmalogen in the heart associated with tafazzin deficiency. Biochemistry. 57:2162-2175. | | | | | |
