# Supplementary material for: Plasmalogen loss caused by remodeling deficiency in mitochondria
Source: Life Sci Alliance. 2019 Aug 21;2(4):e201900348. doi: 10.26508/lsa.201900348 (PMC6707388; doi:10.26508/lsa.201900348)
Supplement: Supplementary file 4 [file LSA-2019-00348_TableS2.docx]

| **Table S2. Contents and Changes (mol %)^1^ of**  **the Liver Phospholipids of the WT versus TAZ-KD Mice** | | | | | |
| --- | --- | --- | --- | --- | --- |
| phospholipid | chemical shift^2^ (ppm) | content | | change in contribution to the total phospholipid | change in terms of the individual content |
|  |  | WT | TAZ-KD | TAZ-KD ‒ WT | [(TAZ-KD ‒ WT)/WT]×100 |
| diacyl PC (+ plasmanylcholine)^3^ | ‒0.177 | 58.6 ± 1.5 | 53.9 ± 0.7 | ‒4.7 ± 1.7 | ‒8.0 ± 2.9 |
| plasmenylcholine | (‒0.166)^4^ | N.R.^5^ | N.R.^5^ | N.A.^7^ | N.A.^7^ |
| PI | 0.032 | 7.8 ± 0.4 | 7.8 ± 1.3 | 0.0 ± 1.4 | ‒0.4 ± 17.4 |
| PS | 0.125 | 3.3 ± 0.4 | 3.3 ± 0.5 | +0.1 ± 0.6 | +1.5 ± 19.3 |
| lyso PC | 0.203 | 2.9 ± 0.8 | 1.3 ± 0.1 | ‒1.6 ± 0.8 | ‒55.4 ± 27.5 |
| diacyl PE (+ plasmanylethanolamine)^3^ | 0.295 | 18.8 ± 1.9 | 25.4 ± 1.7 | +6.6 ± 2.5 | +34.8 ± 13.4 |
| plasmenylethanolamine | 0.333 | 2.5 ± 0.3 | 1.8 ± 0.3 | ‒0.8 ± 0.4 | ‒29.8 ± 16.8 |
| SM | 0.395 | 3.5 ± 0.2 | 3.8 ± 0.1 | +0.2 ± 0.2 | +7.0 ± 6.5 |
| lyso PE | 0.666 | 0.6 ± 0.3 | 0.3 ± 0.0 | ‒0.3 ± 0.3 | ‒56.9 ± 50.4 |
| CL | 0.742 | 1.7 ± 0.1 | 1.7 ± 0.4 | 0.0 ± 0.4 | ‒1.0 ± 20.6 |
| PG | 0.841 | 0.2 ± 0.0 | 0.2 ± 0.0 | 0.0 ± 0.1 | +24.6 ± 30.4 |
| 1-MLCL | 1.005 | N.D.^6^ | 0.2 ± 0.1 | +0.2 ± 0.1 | N.A.^7^ |
| 2-MLCL | 1.130 | N.D.^6^ | 0.3 ± 0.1 | +0.3 ± 0.1 | N.A.^7^ |
| ^1^The average and error, shown as the standard deviation, are obtained from three independent biological samples (N = 3) for each of the WT and TAZ-KD mice.  ^2^Values are from observation at 25°C in 10% (w/v) SDS micellar solution at pH=6.0 [50 mM MES, 50 μM BHT, 10% (v/v) D_2_O], in reference to the diacyl PE (with plasmanylethanolamine) peak set to 0.295 ppm as an internal standard (Kimura et al., 2018).  ^3^The signal of the plasmanyl glycerophospholipid as a minor component overlaps with the signal of the counterpart diacyl glycerophospholipid (Kimura et al., 2018).  ^4^Value estimated based on the difference from that of diacyl PC (+ plasmanylcholine) (Kimura et al., 2018); see footnote 5 in Table S1. The standard deviation of measured chemical shift values of diacyl PC (with plasmanylcholine) in the lipid extract from the liver tissue was 0.004 ppm.  ^5^Signal not resolved.  ^6^Not detected.  ^7^Not applicable.  **Reference**  Kimura, T., A.K. Kimura, M.D. Ren, B. Berno, Y. Xu, M. Schlame, and R.M. Epand. 2018. Substantial decrease in plasmalogen in the heart associated with tafazzin deficiency. Biochemistry. 57:2162-2175. | | | | | |
