# Supplementary material for: Plasmalogen loss caused by remodeling deficiency in mitochondria
Source: Life Sci Alliance. 2019 Aug 21;2(4):e201900348. doi: 10.26508/lsa.201900348 (PMC6707388; doi:10.26508/lsa.201900348)
Supplement: Supplementary file 6 [file LSA-2019-00348_TableS4.docx]

| **Table S4. Contents and Changes (mol %)^1^ of**  **Phospholipids of the Lymphoblast Cells Derived from Healthy Individual Controls and BTHS Patients** | | | | | |
| --- | --- | --- | --- | --- | --- |
| phospholipid | chemical shift^2^ / ppm | content | | change in contribution to the total phospholipid | change in terms of the individual content |
|  |  | control | BTHS | BTHS ‒ control | [(BTHS ‒ control)/control]×100 |
| diacyl PC (+ plasmanylcholine)^3^ | ‒0.162 | 54.5 ± 1.8 | 49.3 ± 1.3 | ‒5.2 ± 2.3 | ‒9.5 ± 4.1 |
| plasmenylcholine | (‒0.151)^4^ | N.R.^5^ | N.R.^5^ | N.A.^7^ | N.A.^7^ |
| PI | 0.044 | 6.1 ± 0.5 | 5.4 ± 0.3 | ‒0.7 ± 0.5 | ‒12.1 ± 8.5 |
| PS | 0.128 | 5.9 ± 0.2 | 5.7 ± 0.4 | ‒0.2 ± 0.5 | ‒3.8 ± 8.0 |
| lyso PC | 0.214 | 0.6 ± 0.1 | 0.3 ± 0.0 | ‒0.3 ± 0.1 | ‒55.0 ± 19.0 |
| diacyl PE (+ plasmanylethanolamine)^3^ | 0.295 | 13.6 ± 1.1 | 21.1 ± 1.4 | +7.5 ± 1.8 | +55.4 ± 12.9 |
| plasmenylethanolamine | 0.335 | 14.4 ± 1.9 | 10.8 ± 1.1 | ‒3.5 ± 2.2 | ‒24.6 ± 15.0 |
| SM | 0.418 | 1.5 ± 0.6 | 2.9 ± 1.2 | +1.4 ± 1.3 | +94.8 ± 87.3 |
| lyso PE | 0.672 | 0.3 ± 0.1 | 0.3 ± 0.1 | 0.0 ± 0.2 | +11.9 ± 55.2 |
| CL | 0.751 | 2.2 ± 0.1 | 0.6 ± 0.1 | ‒1.6 ± 0.1 | ‒73.4 ± 4.9 |
| PG | 0.851 | 0.8 ± 0.3 | 1.6 ± 0.0 | +0.7 ± 0.3 | +86.9 ± 32.8 |
| 1-MLCL | 1.014 | N.D.^6^ | 0.5 ± 0.2 | +0.5 ± 0.2 | N.A.^7^ |
| 2-MLCL | 1.142 | N.D.^6^ | 1.4 ± 0.3 | +1.4 ± 0.3 | N.A.^7^ |
| ^1^The average and error, shown as the standard deviation, are obtained from three independent biological samples (N = 3) for each of healthy individual controls and BTHS patients.  ^2^Values are from observation at 25°C in 10% (w/v) SDS micellar solution at pH=6.0 [50 mM MES, 50 μM BHT, 10% (v/v) D_2_O], in reference to the diacyl PE (with plasmanylethanolamine) peak set to 0.295 ppm as an internal standard (Kimura et al., 2018).  ^3^The signal of the plasmanyl glycerophospholipid as a minor component overlaps with the signal of the counterpart diacyl glycerophospholipid (Kimura et al., 2018).  ^4^Value estimated based on the difference from that of diacyl PC (+ plasmanylcholine) (Kimura et al., 2018); see footnote 5 in Table S1. The standard deviation of measured chemical shift values of diacyl PC (with plasmanylcholine) in the lipid extract from the lymphoblast cells was 0.002 ppm.  ^5^Signal not resolved.  ^6^Not detected.  ^7^Not applicable.  **Reference**  Kimura, T., A.K. Kimura, M.D. Ren, B. Berno, Y. Xu, M. Schlame, and R.M. Epand. 2018. Substantial decrease in plasmalogen in the heart associated with tafazzin deficiency. Biochemistry. 57:2162-2175. | | | | | |
