# Supplementary material for: Plasmalogen loss caused by remodeling deficiency in mitochondria
Source: Life Sci Alliance. 2019 Aug 21;2(4):e201900348. doi: 10.26508/lsa.201900348 (PMC6707388; doi:10.26508/lsa.201900348)
Supplement: Supplementary file 7 [file LSA-2019-00348_TableS5.docx]

| **Table S5. Ratios^1^ of the Protein Expression Level BTHS/Control in Human Lymphoblast, Measured by the Quantitative Western Blotting** | |
| --- | --- |
| Protein | BTHS/Control |
| Pex19p | 1.29 ± 0.03 |
| PMP70 | 0.95 ± 0.01 |
| Far1 | 1.03 ± 0.02 |
| catalase | 1.09 ± 0.49^2^ |
| iPLA_2_*β* | 0.78 ± 0.22 |
| iPLA_2_*γ* | 1.22 ± 0.22 |
| ^1^The average shown as a mean of average values from two independent pairs (i.e., a BTHS‒control pair) of biological samples and each pair with three- or four-replicate WB experiments, and error shown as the standard error of the mean value (N=6 or 7). The statistical analysis was based on a reference (Fay and Gerow, 2013).  ^2^This large number in the error is due to inconsistent trends observed as either an increase or a decrease in the expression level from control to BTHS for the two independent pairs prepared at different times; note that a pair is made to quantify a ratio BTHS/control using the same gel in the WB experiment. One pair gave an increasing trend with a ratio at 1.44 ± 0.08 (N=3), while another gave a decreasing trend at 0.75 ± 0.01 (N=4).  **Reference**  Fay, D.S., and K. Gerow. 2013. A biologist's guide to statistical thinking and analysis. In WormBook. T.C.e.R. Community, editor. WormBook, Pasadena. 1-54. | |
