# Supplementary material for: Plasmalogen loss caused by remodeling deficiency in mitochondria
Source: Life Sci Alliance. 2019 Aug 21;2(4):e201900348. doi: 10.26508/lsa.201900348 (PMC6707388; doi:10.26508/lsa.201900348)
Supplement: Supplementary file 8 [file LSA-2019-00348_Supplementary_Text_3.doc]

Appendix 3

Remodeling of CL acyl chain species by tafazzin: Is there any correlation with abundant species of plasmalogen and the observed plasmalogen loss?

The CL acyl species distribution is known to be very unique with the dominant species of tetralinoleoyl (18:2)_4_ in the mammalian heart (80 mol % in ventricle and 74 mol % in atrium in humans), liver (55 mol % in rat), and kidney (50 mol % in rat) (Schlame et al., 2005; Schlame et al., 2002). The enrichment of CL with a linoleoyl chain appears to relate positively in particular to the NADH-pathway capacity through complex I in the respiratory chain activity (Oemer et al., 2018). In the human lymphoblast, interestingly the dominant species is (18:1)_4_-CL at 32 mol % including significant fractions of both the Δ9 and Δ11 species, and the amount of (18:2)_4_-CL is only 1 mol % (Schlame et al., 2005; Schlame et al., 2002; Xu et al., 2005). Contrastingly, a distribution of CL species in the mammalian brain has been recognized as highly heterogeneous without a dominant species regardless of the presence of tafazzin; (18:2)_4_-CL is very minor in this organ (~2 mol % in dog and ~5 mol % in rat) (Schlame et al., 2002).

As in maintaining the cardiac plasmenylcholine level (Kimura et al., 2018), it is presented in the present report that tafazzin commonly plays an essential role in maintaining the plasmenylethanolamine levels in the other organs and blood cells where ethanolamine is the dominant class of plasmalogen. Let us see if there is any relation between CL, and plasmalogen that is lost in tafazzin deficiency, in their acyl species. In the heart, where the dominant acyl species of CL, a linoleoyl chain, coincides with the dominant acyl species of plasmenylcholine (Arthur et al., 1985; Kikuchi et al., 1999; Schmid and Takahashi, 1968). In the liver and kidney in contrast, the dominant acyl species of CL, likewise a linoleoyl chain, is not enriched in plasmenylethanolamine (Choi et al., 2018). In the brain, plasmenylethanolamine is not enriched either with a linoleoyl chain (Choi et al., 2018; Sun and Horrocks, 1970), while a CL species distribution in this organ being highly diverse (Kiebish et al., 2008).

Key observations in the previous reports that we may consider as an essential factor in enrichment of the (18:2)_4_-CL species in the heart, liver, and kidney are that tafazzin shows a preference in reactivity with phospholipids having an *sn*-2 linoleoyl chain (Abe et al., 2016; Xu et al., 2003; Xu et al., 2006); see discussion in ref. (Kimura et al., 2018). The dominant (18:2)_4_-CL species in the liver and kidney largely reflects tafazzin-catalyzed remodeling probably (i) with diacyl PC which is highly enriched with a linoleoyl chain at the *sn*-2 and (ii) to a lesser degree with diacyl PE which has a minor but significant fraction of a linoleoyl chain at the *sn*-2 (Choi et al., 2018).

Despite the presence of relatively minor fractions of a linoleoyl chain in choline and ethanolamine glycerophospholipids in lymphoblast (2.0 mass % in PC and 1.3 mass % PE) tafazzin enriches this acyl chain to CL at 12.3 mass % although a CL species in the form of (18:2)_4_-CL is only 1 mol %; in BTHS such enrichment of CL with a linoleoyl chain does not occur, and CL contains this species only at 1.4 mass % (Xu et al., 2005).

Extremely low amounts of a linoleoyl species of choline, ethanolamine, and serine glycerophospholipids in the brain have long been recognized (Choi et al., 2018; Martínez and Mougan, 1998; O’Brien et al., 1964; O’Brien and Sampson, 1965). The low amounts of a preferred transacylation species by tafazzin in this organ with the known abundance of arachidonoyl (20:4), docosahexaenoyl (22:6), and oleoyl (18:1) chains in phospholipids (Choi et al., 2018; Martínez and Mougan, 1998; O’Brien et al., 1964; O’Brien and Sampson, 1965) likely contribute to the characteristic presence of diverse CL species (Kiebish et al., 2008; Oemer et al., 2018) that mostly reflect inclusion of those acyl species.

The common losses of plasmalogen due to tafazzin deficiency in the heart, brain, liver, kidney, and lymphoblast, irrespective of the content of a preferred linoleoyl species by the enzyme in those organs and blood cells, suggest that the plasmalogen loss is not associated with its acyl species. Losses of individual plasmalogen species in lymphoblast mitochondria analyzed by MALDI-TOF MS indeed show no relation to a specific species (Fig. S3B). These discussions also favor the plasmalogenase function of cytochrome *c* under oxidative stress as the cause of the plasmalogen loss in tafazzin deficiency, as proposed in the last part of the results section.
