# Supplementary material for: Plasmalogen loss caused by remodeling deficiency in mitochondria
Source: Life Sci Alliance. 2019 Aug 21;2(4):e201900348. doi: 10.26508/lsa.201900348 (PMC6707388; doi:10.26508/lsa.201900348)
Supplement: Supplementary file 9 [file LSA-2019-00348_Supplementary_Text_4.doc]

Appendix 4

Influence of a loss of diacyl PE on expression, organization, and activity of the supercomplexes in yeast

In yeast having diacyl PE deficiency, neither the expression levels of the complexes III and IV, nor the stable assemblies of the yeast forms of the supercomplexes which do not involve complex I, i.e., III_2_IV and III_2_IV_2_, were significantly affected (Baker et al., 2016; Böttinger et al., 2012). However, there were losses in the individual activities of the complexes, dissipation of the membrane potential, as well as a reduction in the cellular ATP level (Baker et al., 2016; Böttinger et al., 2012). Therefore, the loss of diacyl PE negatively influences the activities of complexes III and IV by apparently preserving at least the levels and protein compositions of the supercomplexes.
