# Supplementary material for: Plasmalogen loss caused by remodeling deficiency in mitochondria
Source: Life Sci Alliance. 2019 Aug 21;2(4):e201900348. doi: 10.26508/lsa.201900348 (PMC6707388; doi:10.26508/lsa.201900348)
Supplement: Supplementary file 10 [file LSA-2019-00348_Supplementary_Text_5.doc]

Appendix 5

A loss of plasmenylethanolamine-protein interactions in AD

Contrasting with the relatively minor (5-10%) cases of autosomal dominant AD, the major sporadic AD cases are not associated with gene mutations of either the amyloid precursor protein (APP) or one of the two components of the γ-secretase complex, presenilins 1 and 2, in shifting the APP processing towards formation of amyloid-β_42_. A mitochondrial cascade hypothesis has been proposed and is increasingly invoked to understand the causes of sporadic AD, that include mitochondrial dysfunction due to oxidative stress and a correlation between advancing age and AD risk (Swerdlow et al., 2010; Swerdlow and Khan, 2004). In fact inhibition of the respiratory chain increases tau phosphorylation and also shifts the APP processing to an amyloidogenic derivative (Swerdlow et al., 2010; Swerdlow and Khan, 2004). The oxidative-stress induced plasmalogenase function of cytochrome *c* (Jenkins et al., 2018) is likely relevant to inefficient respiration via a loss of plasmalogen-protein interactions as we discussed in the main text.

Observations of plasmenylethanolamine loss showed anatomic correspondence to neurodegeneration sites in the AD brain (Ginsberg et al., 1995; Han et al., 2001) as well as a correlation with clinical dementia rating (Han et al., 2001). A plasmenylethanolamine loss was observed along with a CL loss in the brain mitochondria of young, 3 month-old 3xTg-AD mice (which express three major genes associated with familial AD, i.e., APPswe, PS1M146V, and tauP301L, and develop the pathological hallmarks in an age dependent manner; Aβ and tau pathologies are detected at 6 and 12 months of age, respectively). These losses of plasmenylethanolamine and CL were accompanied by observations of reductions in the complexes I and IV activities and the ATP level, and an increase of a ratio in the levels of phosphorylated AMP-activated protein kinase (pAMPK) to AMPK in the organ (Monteiro-Cardoso et al., 2015). Interestingly, the loss of CL reflected selective losses of abundant species, largely leveling off differences in the amounts with less abundant species while maintaining the characteristic diversity of CL species in the brain (Monteiro-Cardoso et al., 2015). Thus, the plasmenylethanolamine loss in the AD model took place along with the CL loss and altered distribution of its species. These observations accord with (i) discussion in the current work about a role of plasmenylethanolamine in maintaining the functional integrity of the supercomplexes, and (ii) a report showing that the CL level and remodeling, and the organization of supercomplexes are heavily interdependent (Xu et al., 2016).
