# Supplementary material for: Plasmalogen loss caused by remodeling deficiency in mitochondria
Source: Life Sci Alliance. 2019 Aug 21;2(4):e201900348. doi: 10.26508/lsa.201900348 (PMC6707388; doi:10.26508/lsa.201900348)
Supplement: Supplementary file 11 [file LSA-2019-00348_TableS6.docx]

| **Table S6. List of Antibodies Used in the Quantitative Western Blot Experiments on the Human Lymphoblast Derived from Healthy Individual Controls and BTHS Patients** | | | | | |
| --- | --- | --- | --- | --- | --- |
| Protein | Primary antibody  (Information for Far1 blocking peptide is for the peptide itself) | | | | Secondary antibody^1^ |
|  | Provider | Catalog number | Lot number | Dilution | Dilution |
| Pex19p | Abcam | ab137072 | GR189034-1 | 1:1,000 | 1:3,000 |
| PMP70 | Sigma-Aldrich | P0497 | 014M4751 | 1:200 | 1:3,000 |
| catalase | Thermo Fisher | PA5-23246 | RJ2287941B | 1:2,000 | 1:3,000 |
| Far1 | Aviva Systems Biology | ARP50044_T100 | QC19706-40592 | 1:1,200 | 1:3,000 |
| iPLA_2_*β* | Sigma-Aldrich | SAB4200129 | 040M4767 | 1:1,200 | 1:10,000 |
| iPLA_2_*γ* | Thermo Fisher | PA5-50992 | RI2274612A | 1:1,200 | 1:3,000 |
| Far1 blocking peptide | Aviva Systems Biology | AAP50044 | S27911 | 4.05 μg/ml |  |
| ^1^Goat anti-rabbit IgG-HRP: Provider, Santa Cruz; catalog number, sc-2054; lot number, E1713 | | | | | |
